# Efficient and Accurate Analog Voltage Measurement Using a Direct Sensor-to-Digital Port Interface for Microcontrollers and Field-Programmable Gate Arrays

**DOI:** 10.3390/s24030873

**Published:** 2024-01-29

**Authors:** Marco Grossi

**Affiliations:** Department of Electrical Energy and Information Engineering “Guglielmo Marconi” (DEI), Alma Mater Studiorum–Università di Bologna, Viale del Risorgimento, 2, 40136 Bologna, Italy; marco.grossi8@unibo.it; Tel.: +39-051-2093038

**Keywords:** analog, sensor interfacing, FPGA, embedded system, analog-to-digital converters, signal conversion

## Abstract

Portable sensor systems are usually based on microcontrollers and/or Field-Programmable Gate Arrays (FPGAs) that are interfaced with sensors by means of an Analog-to-Digital converter (ADC), either integrated in the computing device or external. An alternative solution is based on the direct connection of the sensors to the digital input port of the microcontroller or FPGA. This solution is particularly interesting in the case of devices not integrating an internal ADC or featuring a small number of ADC channels. In this paper, a technique is presented to directly interface sensors with analog voltage output to the digital input port of a microcontroller or FPGA. The proposed method requires only a few passive components and is based on the measurements of the duty cycle of a digital square-wave signal. This technique was investigated by means of circuit simulations using LTSpice and was implemented in a commercial low-cost FPGA device (Gowin GW1NR-9). The duty cycle of the square-wave signal features a good linear correlation with the analog voltage to be measured. Thus, a look-up table to map the analog voltage values to the measured duty cycle is not required with benefits in terms of memory occupation. The experimental results on the FPGA device have shown that the analog voltage can be measured with a maximum accuracy of 1.09 mV and a sampling rate of 9.75 Hz. The sampling rate can be increased to 31.35 Hz and 128.31 Hz with an accuracy of 1.61 mV and 2.68 mV, respectively.

## 1. Introduction

Portable and wearable systems for sensing applications have grown many fold in recent years. These systems are usually based on microcontrollers and/or Field Programmable Gate Arrays (FPGAs) and are powered by batteries [1] or scavenge energy from natural sources [2]. The sensing applications cover a wide range of fields, such as environmental monitoring [3,4,5], food screening for quality analysis and safety [6,7,8], biological measurements for human body composition determination and health monitoring [9,10,11], bacterial detection [12,13,14], chemical analysis [15,16,17], health monitoring, and data collection on civil infrastructures [18,19,20].

Sensors are used to measure a physical quantity by a transduction mechanism that outputs an electrical quantity (such as resistance, capacitance, inductance, voltage, or current). Such electrical output is usually converted to an analog voltage and acquired by the sensor system with an Analog-to-Digital Converter (ADC). To guarantee high accuracy in the measured analog voltage, the ADC must be carefully calibrated and a front-end analog interface must be designed for signal conditioning. Xie and Wang, in 2023, presented a digital calibration method for a 10 bit successive approximation register (SAR) ADC that achieves a SNR of 66.9 dB, a power consumption of 70 μW, and a sampling rate of 50 kHz [21]. An ultrafast and low-cost pipelined ADC testing and calibration method was proposed by Chen et al. in 2019 [22]. The proposed technique was implemented on a chip fabricated in 40 nm technology with a power consumption of 10.71 mW and a sampling rate of 125 MHz. Li et al., in 2019, presented a linearity test and calibration method that can be applied to all types of ADCs [23]. A self-testing platform with digital calibration technique for SAR ADCs was proposed by Juan et al. in 2016 that improves SNR with a sampling frequency of 18.75 kHz [24]. Hernández-Gutiérrez et al., in 2023, presented a low-cost embedded system for high-energy radiation detection applications that is able to detect alfa particles and send the nuclear detection events to a server in the cloud [25]. The system is based on an ARM Cortex M4 microcontroller (STM32F407, ST Microelectronics, Geneva, Switzerland) and features an analog circuit for the amplification of the detected photocurrent. A delta-sigma ADC for signal conditioning of automotive piezo-resistive pressure sensors was proposed by Rikan et al. in 2018 [26]. A chip was fabricated through a 0.18 µm CMOS process and achieved over 80 dB SNR with a 2.5 MHz sampling frequency and a power consumption of 646 µW.

A research line in the field of sensor data acquisition is devoted to the study of techniques to directly interface the sensor with the digital port of a microcontroller or FPGA without the use of an ADC. These techniques exploit the fact that the digital input pins of a microcontroller or FPGA integrate a Schmitt trigger circuit to remove the noise present on the input signals, thus the sensor can be interfaced to the digital input port using only a few passive components, without the use of an ADC and analog components for signal processing and filtering. This provides advantages in terms of lower cost, area occupation, and power consumption that are of major interest in the case of battery-operated sensor nodes. Many works from literature cover the case of interfacing with resistive, capacitive, and inductive sensors. Reverter, in 2020, proposed a microcontroller-based interface circuit for non-linear resistive sensors [27]. The same author, in 2022, proposed a direct approach to connect three-wire [28] and four-wire [29] resistive sensors to the digital port of a microcontroller. A technique to directly connect a capacitively coupled resistive sensor with a microcontroller was presented by Areekath et al. in 2020 [30]. Measurement methods for capacitive sensors [31] and lossy capacitive relative humidity sensors [32] based on a direct sensor-to-microcontroller interface circuit were proposed by Czaja in 2020 and 2021. A new type of direct interface circuit for capacitive sensors that is simple in terms of hardware was presented by Hidalgo-López and Castellanos-Ramos in 2022 [33]. A microcontroller-based interface circuit for inductive sensors with variable self-inductance was presented by Kokolanski et al. in 2015 [34]. The same research group, in 2019, proposed a direct interface circuit for differential inductive sensors achieving nonlinearity errors smaller than 1% and an overall measuring time of a few milliseconds [35]. A resolution enhancement method for direct interfacing of inductive sensors was proposed by Asif et al. in 2018 [36].

In the case of sensors featuring an analog output voltage, works from the literature also present the sensor direct interface. Peter et al. in 1998 presented a technique to measure an analog voltage with a microcontroller without an integrated ADC [37]. The proposed method requires the presence of an analog comparator (either integrated in the microcontroller or external) and was validated on a Microchip PIC16 microcontroller featuring an integrated analog comparator. This technique was implemented in different microcontrollers and FPGAs. Soldera et al., in 2005, presented the implementation on the HC9S08Rx microcontroller family (NXP Semiconductors, Eindhoven, Netherlands) of a 10 bit first-order continuous-time delta-sigma ADC using an integrated analog comparator [38]. Weber and Windish, in 2007, presented the implementation on a MSP430 microcontroller (Texas Instruments, Dallas, TX, USA) for the measurement of an analog voltage for industrial applications using an external delta-sigma modulator AD7400 (Analog Devices, Wilmington, MA, USA) [39]. In 2011, the technique was implemented on a Altera Cyclone IV FPGA device with an integrated analog comparator and support for low voltage differential signals (LVDSs) [40]. A significant improvement over the previous designs was proposed by Bengtsson in 2012 [41]. In this case, the property in which digital input pins of a microcontroller or FPGA behave like a Schmitt trigger circuit was exploited to remove the need for an analog comparator. The proposed technique was tested on a Microchip PIC18F458 microcontroller (Microchip Technology, Chandler, AZ, USA), and the results showed that an analog voltage can be determined with a resolution equivalent to a 12 bit ADC and a sampling rate of 65 Hz. The main drawbacks of the proposed method are the limited bandwidth and the high cost in terms of memory occupation. In fact, before the measurement, the capacitor must be charged to the analog voltage to be estimated, and this requires a significant amount of time, with a negative impact on the sampling rate. Moreover, the characteristic exploited to estimate the analog voltage is significantly non-linear. Thus, to guarantee the 12 bit ADC accuracy, a look-up table (LUT) must be used to map the analog voltage values to the measurements carried out with the microcontroller counter, and this results in high memory occupation that can be unacceptable, in particular in the case of a low-cost device featuring small on-chip memory.

In this paper, a technique is presented for the measurement of an analog voltage without an ADC that can be implemented on low-cost microcontrollers and FPGAs. While most microcontrollers, nowadays, integrate an internal ADC, some devices feature a low number of ADC channels. For example, the RP2040 is a 32 bit dual ARM Cortex-M0+ microcontroller present in the Raspberry Pi Pico development board that features only a four-channel 12 bit ADC [42]. In such situations, the proposed technique can be used if the project needs a number of ADC channels higher than that available. In the case of FPGAs, some low-cost devices do not even feature an integrated ADC. The proposed technique uses the same external circuit of the work of Bengtsson [41] but improves it by providing a much more linear characteristic to estimate the analog voltage and removing the initial capacitance charge step that negatively affects the sampling rate. The feasibility and performance of the proposed technique were evaluated by means of LTSpice simulations and tested on a low-cost commercial FPGA. In Section 2, the working principle of the proposed technique is discussed. In Section 3, the results of LTSpice simulations are presented to validate the feasibility of the proposed technique and estimate the maximum performance in terms of accuracy and sampling rate. In Section 4, the implementation of the proposed technique on a commercial low-cost FPGA device is presented, and in Section 5, the results of measurements obtained with the FPGA device are discussed. In Section 6, the performance of the proposed technique is compared with similar techniques from literature and a 12 bit ADC integrated within a low-cost microcontroller. Finally, concluding remarks are presented in Section 7.

## 2. Measurement Technique

A measurement technique to estimate an analog voltage without the use of an ADC is presented. First, in Section 2.1, the working principle of the proposed technique is discussed. Then, in Section 2.2, the parameters of the digital interface are measured and presented for different commercial devices (microcontrollers and FPGAs). Finally, in Section 2.3, circuits are presented that can be used to change the values of the digital interface parameters.

### 2.1. Working Principle

**Figure 1:**
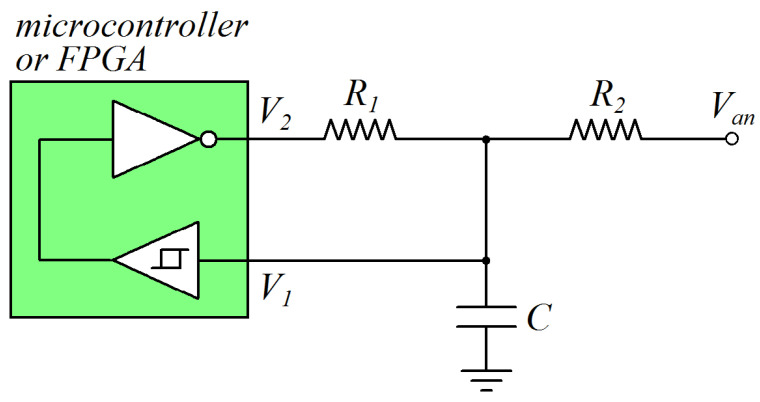
Schematic of the circuit used to implement the proposed technique for analog voltage measurements without an ADC.

**Figure 2:**
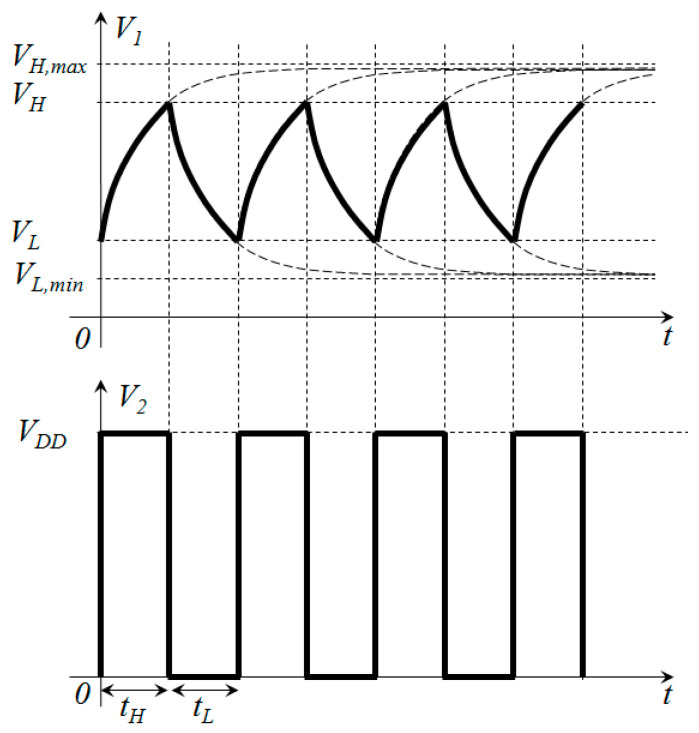
Waveforms of the voltage signals for the circuit presented in Figure 1.

The schematic of the circuit used to implement the proposed technique is shown in Figure 1, where *V_an_* represents the analog voltage to be measured. The circuit is composed of three passive components, two resistances, *R*_1_ and *R*_2_, a capacitance, *C*, and a computing device (microcontroller or FPGA). The Schmitt trigger circuit (with thresholds *V_H_* and *V_L_*) and the digital not gate (implemented in the programmable logic in a FPGA or in software in a microcontroller) are both integrated into the computing device. The working principle of the circuit presented in Figure 1 is shown in Figure 2, where the waveforms of the voltages *V*_1_ and *V*_2_ are plotted vs time. The circuit works as an astable multivibrator: when the voltage *V*_1_ increases over *V_H_*, the output voltage *V*_2_ switches from *V_DD_* to 0 and the capacitance *C* discharges; thus, *V*_1_ decreases with time. When the voltage *V*_1_ decreases under *V_L_*, the output voltage *V*_2_ switches from 0 to *V_DD_* and the capacitance *C* charges; thus, *V*_1_ increases with time. The output voltage *V*_2_ is a pulse width modulated (PWM) signal, with *t_H_* being the time when *V*_2_ = *V_DD_* and *t_L_* being the time when *V*_2_ = 0.

When *V*_1_ increases over *V_H_* and *V*_2_ switches from *V_DD_* to 0, the circuit is modeled with the following equations:

(1) $$C\frac{dV_{1}}{dt}=-\frac{V_{1}}{R_{1}}+\frac{V_{an}-V_{1}}{R_{2}}=-\frac{R_{1}+R_{2}}{R_{1}R_{2}}V_{1}+\frac{V_{an}}{R_{2}}$$

Assuming the analog voltage *V_an_* is constant during the measurement, the differential Equation (1) can be integrated with the initial condition *V*_1_(0) = *V_H_*, and *V*_1_(*t*) can be calculated as

(2) $$V_{1}\left(t\right)=\frac{R_{1}}{R_{1}+R_{2}}V_{an}+\left(V_{H}-\frac{R_{1}}{R_{1}+R_{2}}V_{an}\right)e^{-\frac{R_{1}+R_{2}}{R_{1}R_{2}C}t}$$

Now, imposing the condition *V*_1_(*t_L_*) = *V_L_*, the time interval *t_L_* can be calculated as

(3) $$t_{L}=\frac{R_{1}R_{2}C}{R_{1}+R_{2}}\log\frac{V_{H}-\frac{R_{1}}{R_{1}+R_{2}}V_{an}}{V_{L}-\frac{R_{1}}{R_{1}+R_{2}}V_{an}}$$

As can be seen in Equation (2), the voltage *V*_1_ is *V_H_* at the beginning of the capacitor discharge, and it asymptotically converges to *V_an_∙R*_1_/(*R*_1_ + *R*_2_) as time → +∞. Thus, the following constraint must be valid:

(4) $$V_{L}>\frac{R_{1}}{R_{1}+R_{2}}V_{an}$$

This constraint must be valid for all analog voltages *V_an_* in the range 0–*V_DD_*, and thus,

(5) $$V_{L}>V_{L,inf}=\frac{R_{1}}{R_{1}+R_{2}}V_{DD}$$

When *V*_1_ decreases under *V_L_* and *V*_2_ switches from 0 to *V_DD_*, the circuit is modeled with the following equations:

(6) $$C\frac{dV_{1}}{dt}=\frac{V_{DD}-V_{1}}{R_{1}}+\frac{V_{an}-V_{1}}{R_{2}}=-\frac{R_{1}+R_{2}}{R_{1}R_{2}}V_{1}+\frac{V_{an}}{R_{2}}+\frac{V_{DD}}{R_{1}}$$

Assuming the analog voltage *V_an_* is constant during the measurement, the differential Equation (6) can be integrated with the initial condition *V*_1_(0) = *V_L_*, and *V*_1_(*t*) can be calculated as

(7) $$V_{1}\left(t\right)=\frac{R_{1}}{R_{1}+R_{2}}V_{an}+\frac{R_{2}}{R_{1}+R_{2}}V_{DD}+\left(V_{L}-\frac{R_{1}}{R_{1}+R_{2}}V_{an}-\frac{R_{2}}{R_{1}+R_{2}}V_{DD}\right)e^{-\frac{R_{1}+R_{2}}{R_{1}R_{2}C}t}$$

Now, imposing the condition *V*_1_(*t_H_*) = *V_H_*, the time interval *t_H_* can be calculated as

(8) $$t_{H}=\frac{R_{1}R_{2}C}{R_{1}+R_{2}}\log\frac{V_{L}-\frac{R_{1}}{R_{1}+R_{2}}V_{an}-\frac{R_{2}}{R_{1}+R_{2}}V_{DD}}{V_{H}-\frac{R_{1}}{R_{1}+R_{2}}V_{an}-\frac{R_{2}}{R_{1}+R_{2}}V_{DD}}$$

As can be seen in Equation (7), the voltage *V*_1_ is *V_L_* at the beginning of the capacitor charge and it asymptotically converges to *V_an_∙R*_1_/(*R*_1_ + *R*_2_) + *V_DD_∙R*_2_/(*R*_1_ + *R*_2_) as time → +∞. Thus, the following constraint must be valid:

(9) $$V_{H}<\frac{R_{1}}{R_{1}+R_{2}}V_{an}+\frac{R_{2}}{R_{1}+R_{2}}V_{DD}$$

This constraint must be valid for all analog voltages *V_an_* in the range 0–*V_DD_*, and thus,

(10) $$V_{H}<V_{H,sup}=\frac{R_{2}}{R_{1}+R_{2}}V_{DD}$$

As can be seen from Equations (3) and (8), the time intervals *t_L_* and *t_H_* are non-linear functions of the analog voltage *V_an_*, the digital interface parameters *V_L_* and *V_H_*, and the circuit components (*R*_1_, *R*_2_ and *C*). Moreover, since the circuit of Figure 1 must work as an astable multivibrator, the conditions defined in Equations (5) and (10) must be valid. 

### 2.2. Digital Interface Parameters in Commercial Devices

Since the time intervals *t_L_* and *t_H_* are functions of the digital interface parameters *V_L_* and *V_H_*, the values of such parameters were measured for different commercial microcontrollers and FPGAs. The measurements were carried out using a Nucleo L073RZ development board (ST Microelectronics, Geneva, Switzerland) that embeds on board the STM32L073RZT6 microcontroller with an integrated 12 bit DAC (measurement unit, MU). The microcontroller was programmed to generate a triangular voltage signal on the DAC output (0–3.3 V, with steps of 0.806 mV and an inter-steps delay of 100 ms), and this voltage signal was provided to the digital input pin of the device under testing (DUT). The digital value read by the DUT is replicated on a digital output pin that is given as input to a digital input pin of the MU. For each DAC voltage step, the value of the analog voltage and the digital value read by the MU are sent to a laptop PC using UART, and the values of the digital interface parameters are calculated. For each DUT, five different digital input pins were tested and each measurement was carried out in triplicate.

The measured values of *V_L_* and *V_H_* for different microcontrollers and FPGAs are reported in Table 1. For each device, the reported data are the average values of *V_H_* and *V_L_*, *V_t_* = (*V_H_* + *V_L_*)/2, Δ*V* = *V_H_* − *V_L_*, and the coefficient of variation of Δ*V* (*CV*_Δ*V*_) is defined as the ratio between the standard deviation and the mean value of Δ*V*. 

The results of Table 1 show that the digital interface parameters are strongly dependent on the technology, with different devices characterized by significantly different values of *V_L_* and *V_H_*. Regarding the intra-device variability of the digital interface parameters, the results have shown that the parameters *V_L_* and *V_H_* for different input pins of the same device follow a Gaussian distribution with differences that can be up to 7.5% of the average values of the interface parameters. Thus, for accurate measurements, the selected pin must be calibrated by measuring the specific values of the parameters *V_L_* and *V_H_*. From preliminary simulations, it was also assessed that the interface parameters are affected by the power supply voltage and temperature. Then, the system must feature voltage regulators to provide a stable power supply and capacitors close to the power supply pins to decouple the high-frequency noise generated by the internal circuitry. Regarding the variation with temperature, *V_L_* and *V_H_* decreases linearly with increasing temperature values, with an average change rate of 1.24 mV/°C in the case of *V_H_* and 0.36 mV/°C in the case of *V_L_*. Thus, if measurements are carried out in the presence of significant temperature fluctuations (>5 °C), a temperature sensor can be used to compensate for the variation of the interface parameters with temperature. 

### 2.3. Circuits to Change the Digital Interface Parameters

The data reported in Table 1 show that different devices are characterized by different values for the digital interface parameters. In particular, the FPGA from the Basys 3 development board [45] is characterized by a small value of Δ*V* (about 52 mV) that cannot be compatible with accurate *V_an_* measurements if the signal-to-noise ratio (SNR) is not sufficiently high. Similarly, the parameter *V_t_* can assume values significantly different from *V_DD_*/2 = 1.65 V, and this, as will be explained in Section 3, results in lower accuracy of the measured *V_an_* value. To solve these problems, a couple of circuits were proposed that can change the values of parameters *V_t_* and Δ*V* at the cost of a limited increase of the interface circuit complexity.

**Figure 3:**
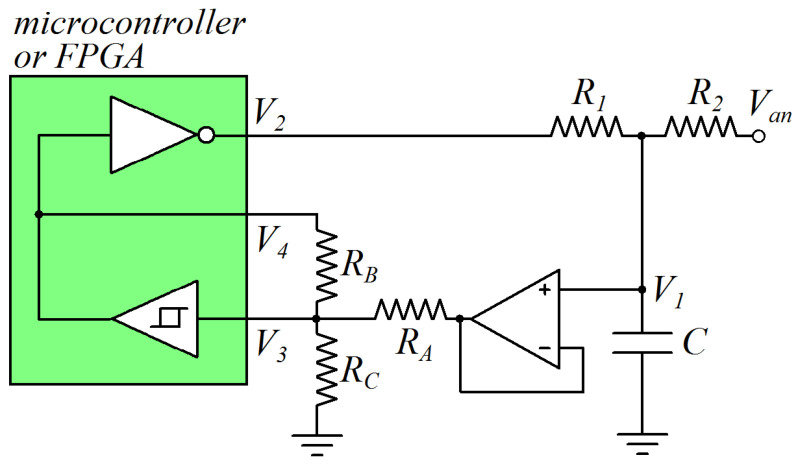
Circuit for the modulation of digital interface parameters in the case of a DUT with *V_t_* < *V_DD_*/2.

The first circuit, presented in Figure 3, can be used to change the digital interface parameters in the case the value of *V_t_* is lower than *V_DD_*/2. The operational amplifier working in voltage follower mode is used to provide a high input impedance for the voltage signal *V*_1_. Indicating that when *V_L_* and *V_H_* are the digital interface parameters of the device (at node 3) and when V_L_* and V_H_* are the digital interface parameters at node 1, it is

(11) $${V_{L}}^{*}=\left(1+\frac{R_{A}}{R_{B}}+\frac{R_{A}}{R_{C}}\right)V_{L}-\frac{R_{A}}{R_{B}}V_{DD}$$

(12) $${V_{H}}^{*}=\left(1+\frac{R_{A}}{R_{B}}+\frac{R_{A}}{R_{C}}\right)V_{H}$$

Thus, the values of V_t_* and ΔV* can be calculated as

(13) $${V_{t}}^{*}=\frac{{V_{H}}^{*}+{V_{L}}^{*}}{2}=\left(1+\frac{R_{A}}{R_{B}}+\frac{R_{A}}{R_{C}}\right)V_{t}-\frac{R_{A}}{R_{B}}\frac{V_{DD}}{2}$$

(14) $$\Delta V^{*}={V_{H}}^{*}-{V_{L}}^{*}=\left(1+\frac{R_{A}}{R_{B}}+\frac{R_{A}}{R_{C}}\right)\Delta V+\frac{R_{A}}{R_{B}}V_{DD}$$

By choosing appropriate values of *R_A_*, *R_B_*, and *R_C_*, the selected values of V_L_*, V_H_*, V_t_*, and ΔV* can be obtained.

**Figure 4:**
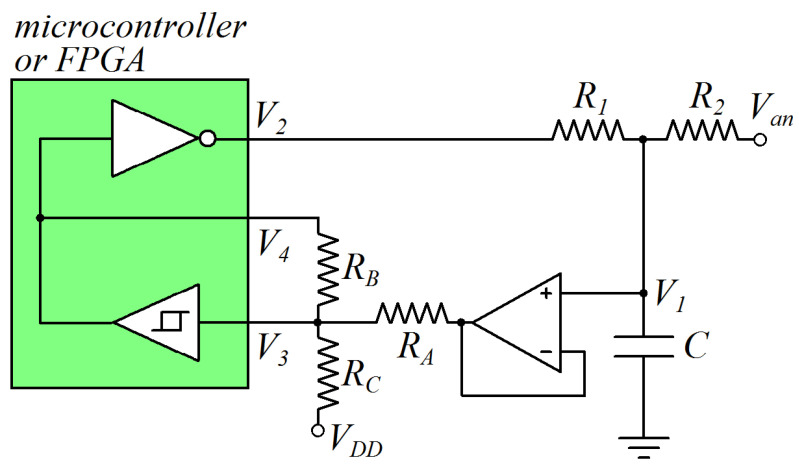
Circuit for the modulation of digital interface parameters in the case of a DUT with *V_t_* > *V_DD_*/2.

Similarly, in the case of a device characterized by *V_t_* > *V_DD_*/2, the digital interface parameters can be changed using the circuit of Figure 4. Indicating that when *V_L_* and *V_H_* are the digital interface parameters of the device (at node 3) and when V_L_* and V_H_* are the digital interface parameters at node 1, they are:

(15) $${V_{L}}^{*}=\left(1+\frac{R_{A}}{R_{B}}+\frac{R_{A}}{R_{C}}\right)V_{L}-\left(\frac{R_{A}}{R_{B}}+\frac{R_{A}}{R_{C}}\right)V_{DD}$$

(16) $${V_{H}}^{*}=\left(1+\frac{R_{A}}{R_{B}}+\frac{R_{A}}{R_{C}}\right)V_{H}-\frac{R_{A}}{R_{C}}V_{DD}$$

Thus, the values of V_t_* and ΔV* can be calculated as:

(17) $${V_{t}}^{*}=\frac{{V_{H}}^{*}+{V_{L}}^{*}}{2}=\left(1+\frac{R_{A}}{R_{B}}+\frac{R_{A}}{R_{C}}\right)V_{t}-\frac{R_{A}}{R_{C}}V_{DD}-\frac{R_{A}}{R_{B}}\frac{V_{DD}}{2}$$

(18) $$\Delta V^{*}={V_{H}}^{*}-{V_{L}}^{*}=\left(1+\frac{R_{A}}{R_{B}}+\frac{R_{A}}{R_{C}}\right)\Delta V+\frac{R_{A}}{R_{B}}V_{DD}$$

Additionally, in this case, by choosing appropriate values of *R_A_*, *R_B_*, and *R_C_*, the selected values of V_L_*, V_H_*, V_t_*, and ΔV* can be obtained.

Simulations were carried out to evaluate the impact of the accuracy of the resistors *R_A_*, *R_B_*, and *R_C_* on the accuracy of the parameters V_L_* and V_H_*. The results have shown that, in the presence of 10% variations in *R_A_*, *R_B_*, and *R_C_*, the corresponding variations in V_L_* and V_H_* are 2.72% and 4.44% for variations in *R_A_*, 2.11% and 0.7% for variations in *R_B_*, 4.71% and 3.52% for variations in *R_C_*. 

## 3. Circuit Simulations

To test the feasibility and performance of the proposed technique to estimate the value of analog voltages without an ADC, circuit simulations were carried out using the software LTSpice XVII (Analog Devices, Wilmington, MA, USA) [48]. The circuit presented in Figure 1 was simulated by modeling the digital interface of the DUT using a Schmitt trigger circuit with suitable values of *V_L_* and *V_H_*. Initially, the case study of a digital interface characterized by *V_t_* = 1.65 V, Δ*V* = 0.5 V, *V_L_* = 1.4 V, and *V_H_* = 1.9 V is considered, and the results are presented in Section 3.1. Next, the case study of digital interfaces characterized by *V_t_* = 1.65 V and multiple values of Δ*V* is considered and the results are presented in Section 3.2. Then, the case study of digital interfaces characterized by Δ*V* = 0.5 V and multiple values of *V_t_* is considered, and the results are presented in Section 3.3. Finally, the maximum performance and accuracy of the proposed technique are estimated in Section 3.4.

### 3.1. Digital Interface with V_t_ = 1.65 V and *Δ*V = 0.5 V

In this section, the circuit of Figure 1 is simulated in the case where the digital interface is characterized by the parameters *V_t_* = 1.65 V, Δ*V* = 0.5 V, *V_L_* = 1.4 V, and *V_H_* = 1.9 V. Four different parameters of the output signal *V*_2_ are considered: the period *T* = *t_H_* + *t_L_*, the time interval *t_H_* when *V*_2_ = *V_DD_*, the time interval *t_L_* when *V*_2_ = 0, and the duty cycle (*DC*), defined as

(19) $$DC=100\frac{t_{H}}{t_{H}+t_{L}}$$

Simulations were carried out with *V_DD_* = 3.3 V, *R*_1_ = 10 kΩ, and different values of the capacitance *C* (320 nF, 150 nF, 60 nF, 47 nF, 32 nF, 20 nF, 14 nF, 9 nF, 5 nF, 2 nF, and 1 nF). Since the value of the capacitance *C* affects the switching frequency of the signal *V*_2_ but does not affect the linearity of the achieved characteristics, the results of the simulations are reported for the case *C* = 60 nF. Under these conditions, the resistance *R*_2_ must comply with the constraints defined by Equations (5) and (10), and thus,

(20) $$R_{2}>R_{1}\frac{V_{DD}-V_{L}}{V_{L}}=13.57\;k\Omega$$

(21) $$R_{2}>R_{1}\frac{V_{H}}{V_{DD}-V_{H}}=13.57\;k\Omega$$

**Figure 5:**
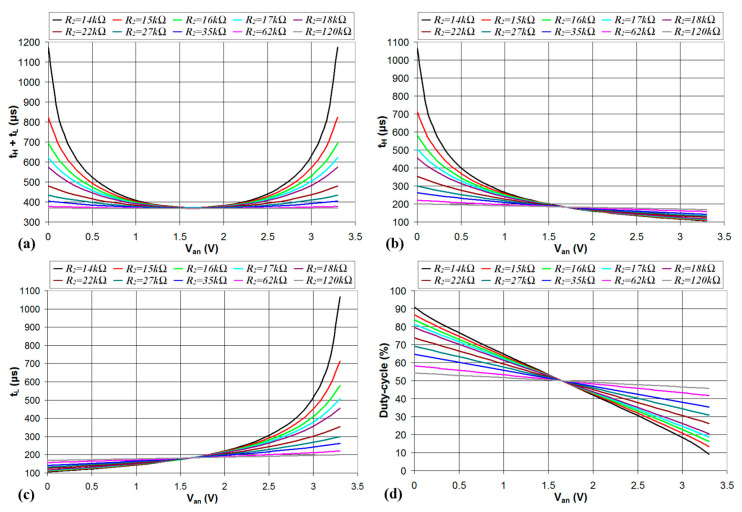
Simulated values of period (**a**), *t_H_* (**b**), *t_L_* (**c**), and duty cycle (**d**) of the output signal *V*_2_ plotted vs. the analog voltage *V_an_* for *R*_1_ = 10 kΩ, *C* = 60 nF, and different values of *R*_2_.

**Figure 6:**
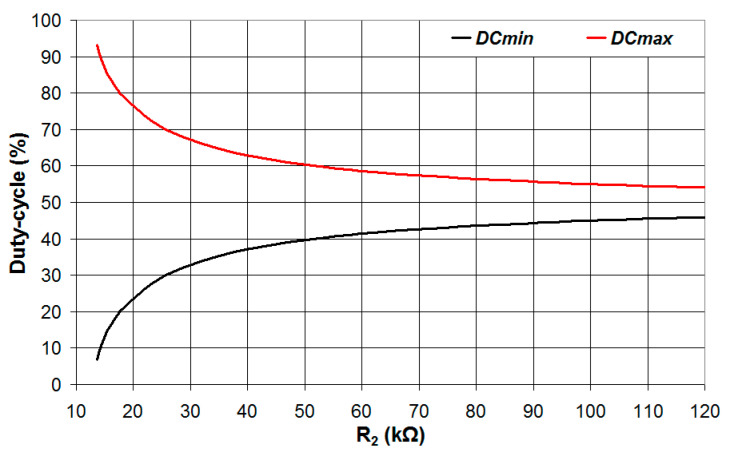
Minimum and maximum values of the duty cycle plotted vs. the value of the resistance *R*_2_.

**Figure 7:**
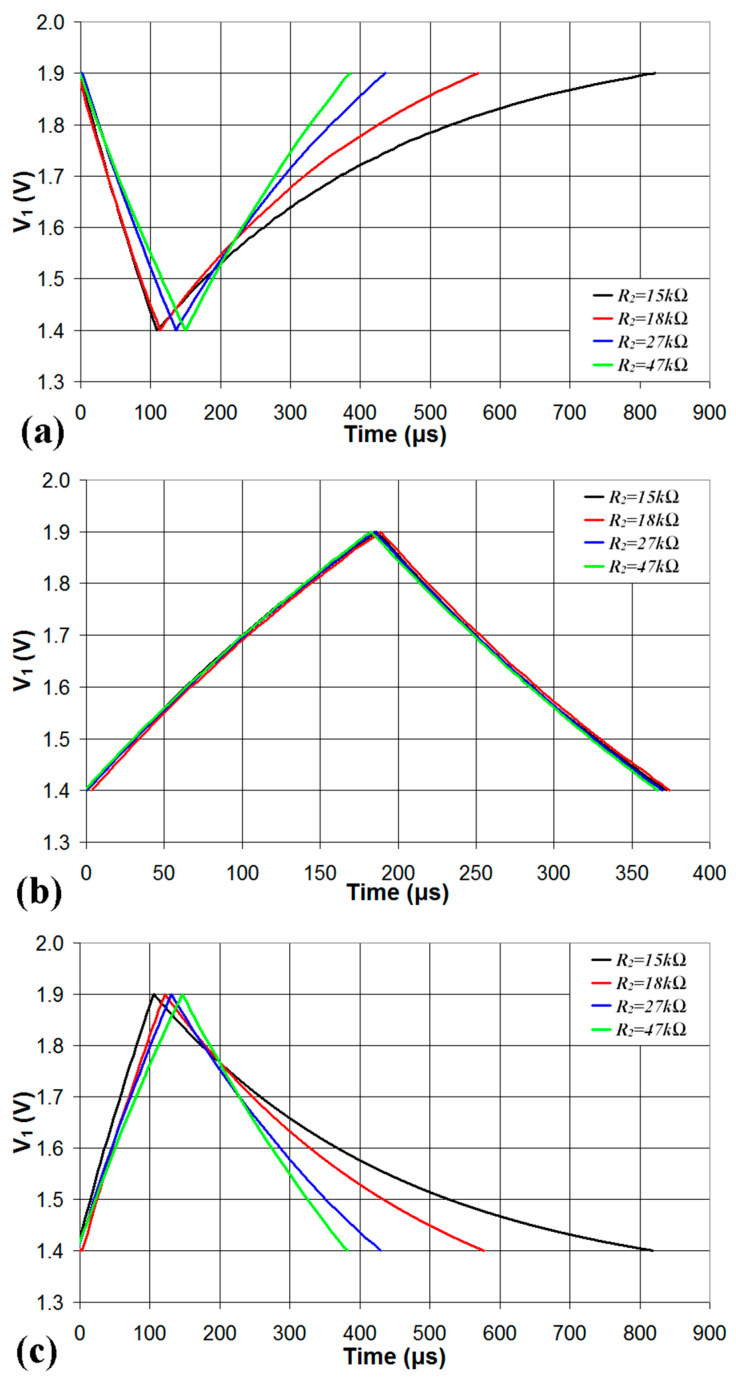
Waveforms of the signal *V*_1_ plotted vs. time during the charging and discharging phase of the capacitor *C* for four different values of *R*_2_ in the case of (**a**) *V_an_* = 0, (**b**) *V_an_* = *V_DD_*/2 = 1.65 V, and (**c**) *V_an_* = *V_DD_* = 3.3 V.

Since *V_t_* = *V_DD_*/2 = 1.65 V, Equations (20) and (21) provide the same constraint *R*_2_ > 13.57 kΩ. Simulations were carried out for different values of *R*_2_, and the results are reported in Figure 5. As can be seen, the period of *V*_2_, shown in Figure 5a, is a non-monotonous function of *V_an_*, and thus, it cannot be used to estimate the value of the analog voltage. The time intervals *t_H_* and *t_L_*, shown in Figure 5b and c, respectively, are monotonous with *V_an_* but, as also presented in Equations (3) and (8), are nonlinear functions of *V_an_*. The exploitation of *t_H_* and *t_L_* to estimate *V_an_*, albeit feasible, must use a look-up table to map the values of *V_an_* to the measured values of *t_H_* and *t_L_* with significant use of the device memory. Moreover, the resolution of the estimated *V_an_* is not uniform in the analog voltage range (0–3.3 V) but is higher for low values of *V_an_* in the case of *t_H_* and high values of *V_an_* in the case of *t_L_*. In the case of the duty cycle, shown in Figure 5d, the value is decreasing with *V_an_*, and the relation between the duty cycle and *V_an_* is linear; thus, the analog voltage *V_an_* can be estimated from the measured duty cycle without the need to use a look-up table to map the values of *V_an_* to the duty cycle. Moreover, the duty-cycle range increases for decreasing values of *R*_2_. This is clearly shown in Figure 6, where the maximum and minimum values of the duty cycle (achieved for *V_an_* = 0 and *V_an_* = 3.3 V, respectively) are plotted vs. *R*_2_. The maximum duty cycle increases and the minimum duty cycle decreases, in particular, when *R*_2_ approaches the lower limit defined by the constraints of Equations (20) and (21). The relation between the duty cycle and *R*_2_ for different values of *V_an_* can be explained by considering the charging and discharging times of the capacitor *C*, as shown in Figure 7.

In Figure 7a, the voltage *V*_1_ is plotted vs. time in the case *V_an_* = 0 for four different values of *R*_2_; while the discharging time *t_L_* is marginally affected by *R*_2_, the charging time *t_H_* strongly increases with the decrease of *R*_2_ (thus increasing the maximum value of the duty cycle). In Figure 7b, the voltage *V*_1_ is plotted vs. time in the case *V_an_* = *V_DD_*/2 = 1.65 V for four different values of *R*_2_; in this case, both the charging time *t_H_* and the discharging time *t_L_* are marginally affected by *R*_2_ and the value of the duty cycle is close to 50%. In Figure 7c, the voltage *V*_1_ is plotted vs. time in the case *V_an_* = *V_DD_* = 3.3 V for four different values of *R*_2_; while the charging time *t_H_* is marginally affected by *R*_2_, the discharging time *t_L_* strongly increases with the decrease of *R*_2_ (thus decreasing the minimum value of the duty cycle).

**Figure 8:**
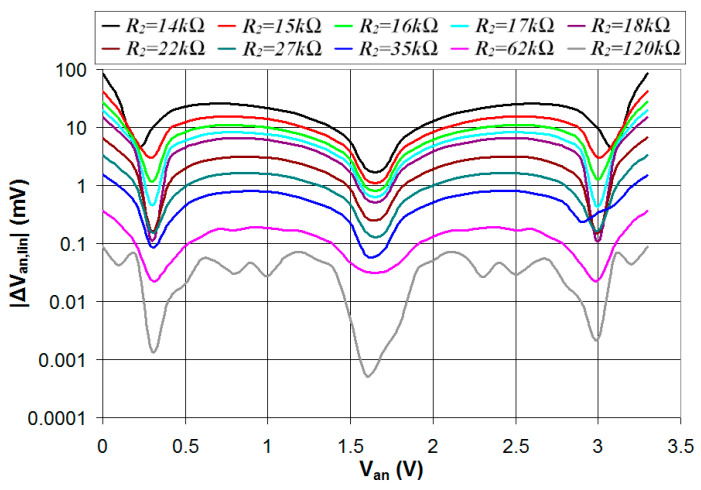
Absolute value of the error in the estimated *V_an_* plotted vs. the real value of *V_an_* for different values of the resistance *R*_2_.

While the duty-cycle range (and thus the sensitivity of *V_an_*) increases for lower values of *R*_2_, the linearity of the relation between the duty cycle and *V_an_* decreases as well. This is shown in Figure 8, where the absolute value of the error in the estimated *V_an_* (|Δ*V_an_*_,*lin*_|), the difference between *V_an_* and the value estimated from the duty cycle using the linear regression line, is plotted vs. *V_an_* for different values of the resistance *R*_2_. As can be seen, the increased linearity achieved for higher values of *R*_2_ results in a lower error in the estimated value of *V_an_*. In particular, for *R*_2_ = 120 kΩ, the estimated error |Δ*V_an_*_,*lin*_| is lower than 1 mV and comparable to the resolution of a 12 bit ADC with a 0–3.3 V input range.

### 3.2. Digital Interface with V_t_ = 1.65 V and *Δ*V Variable

**Figure 9:**
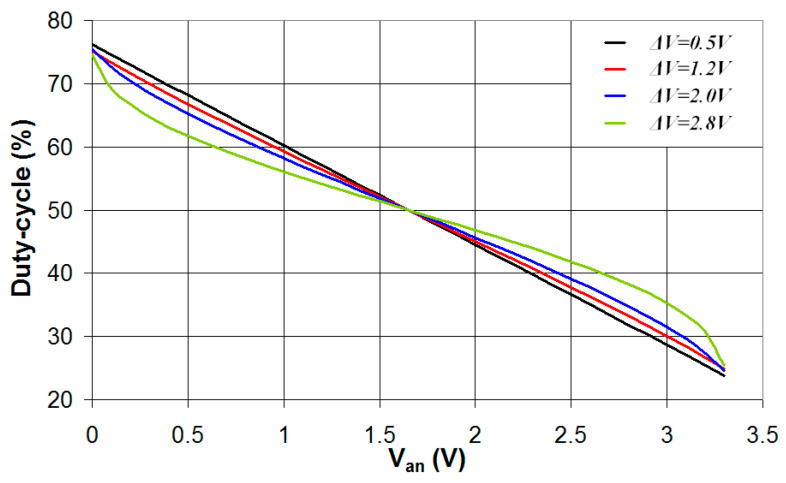
Simulated values of the duty cycle of the output signal *V*_2_ plotted vs. the analog voltage *V_an_* in the case *V_t_* = *V_DD_*/2 = 1.65 V and four different values of Δ*V*.

In this section, the circuit of Figure 1 is simulated in the case where the digital interface is characterized by the parameters *V_t_* = 1.65 V and four different values of Δ*V* (0.5 V, 1.2 V, 2.0 V, 2.8 V). The simulations were carried out with values of *R*_2_ that results in comparable values of the duty-cycle range for *V_an_* values in the range 0–3.3 V. In particular, *R*_2_ = 20 kΩ in the case Δ*V* = 0.5 V (*DC_min_* = 23.7%, *DC_max_* = 76.3%), *R*_2_ = 26 kΩ in the case Δ*V* = 1.2 V (*DC_min_* = 24.8%, *DC_max_* = 75.1%), *R*_2_ = 43 kΩ in the case Δ*V* = 2.0 V (*DC_min_* = 24.6%, *DC_max_* = 75.4%), and *R*_2_ = 123 kΩ in the case Δ*V* = 2.8 V (*DC_min_* = 25.5%, *DC_max_* = 74.5%). The results of the simulations are presented in Figure 9, where the duty cycle is plotted vs. *V_an_*. As can be seen, in the cases Δ*V* = 0.5 V and Δ*V* = 1.2 V the duty cycle is a quasi-linear function of the analog voltage *V_an_*, while for higher values of Δ*V*, significant deviation from the linear response is present. The analog voltage *V_an_* was estimated from the value of the duty cycle using the linear regression line fitting the curves presented in Figure 9, and the absolute value of the error in the estimated *V_an_* (|Δ*V_an_*_,*lin*_|) is plotted vs. *V_an_* for the four different values of Δ*V*. 

**Figure 10:**
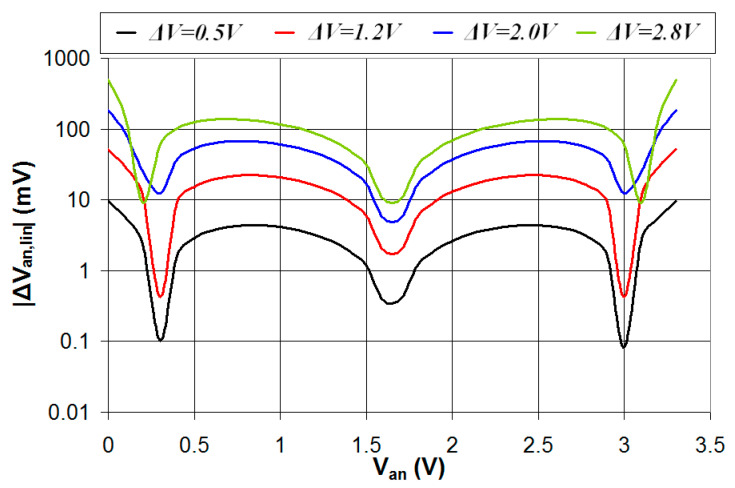
Absolute value of the error in the estimated *V_an_* plotted vs. the real value of *V_an_* for *V_t_* = *V_DD_*/2 = 1.65 V and four different values of Δ*V*.

The results, presented in Figure 10, show how smaller values of Δ*V* are characterized by a better linear response of the relation between the duty cycle and *V_an_* and lower values of |Δ*V_an_*_,*lin*_|. Thus, to achieve good accuracy in the estimation of *V_an_*, a suitable value of Δ*V* must be considered that must be not too high to provide a quasi-linear response but also not too low to guarantee a reliable operation of the circuit depending on the signal-to-noise ratio (SNR). 

### 3.3. Digital Interface with V_t_ Variable and *Δ*V = 0.5 V

**Figure 11:**
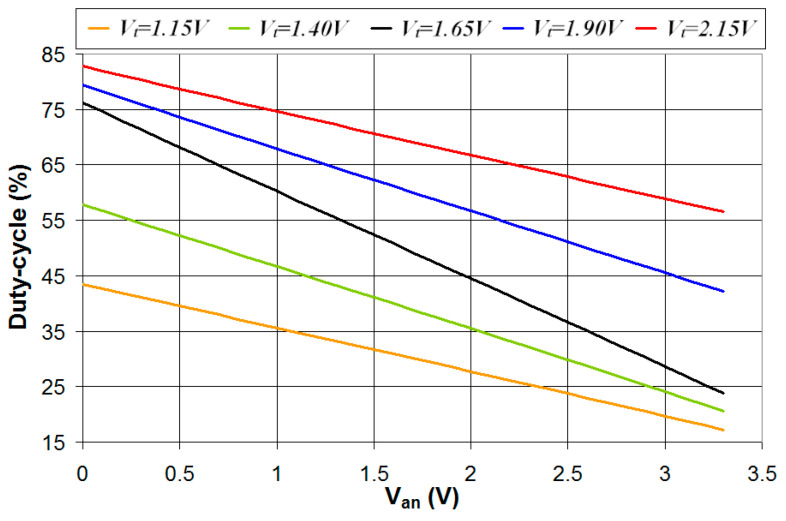
Simulated values of the duty cycle of the output signal *V*_2_ plotted vs. the analog voltage *V_an_* in the case of five different values of *V_t_* and Δ*V* = 0.5 V.

In this section, the circuit of Figure 1 is simulated in the case where the digital interface is characterized by five different values of *V_t_* (1.15 V, 1.40 V, 1.65 V, 1.90 V, and 2.15 V) and Δ*V* = 0.5 V. The digital interface parameters are, respectively, *V_L_* = 0.90 V and *V_H_* = 1.40 V for *V_t_* = 1.15 V, *V_L_* = 1.15 V, and *V_H_* = 1.65 V for *V_t_* = 1.40 V, *V_L_* = 1.40 V, and *V_H_* = 1.90 V for *V_t_* = 1.65 V, *V_L_* = 1.65 V, and *V_H_* = 2.15 V for *V_t_* = 1.90 V, *V_L_* = 1.90 V, and *V_H_* = 2.40 V for *V_t_* = 2.15 V. The simulations were carried out with *R*_2_ = 20 kΩ for *V_t_* = 1.65 V, *R*_2_ = 28 kΩ for *V_t_* = 1.40 V and *V_t_* = 1.90 V, and *R*_2_ = 40 kΩ for *V_t_* = 1.15 V and *V_t_* = 2.15 V. The results of the simulations are presented in Figure 11. As can be seen, the case *V_t_* = 1.65 V is characterized by the highest range of the duty cycle and, thus, the highest sensitivity in the estimation of *V_an_*. The reason for such behavior can be explained by considering the constraint conditions defined by Equations (5) and (10): it is *V_L_*_,*inf*_ = 1.1 V and *V_H_*_,*sup*_ = 2.2 V for *R*_2_ = 20 kΩ, *V_L_*_,*inf*_ = 0.87 V and *V_H_*_,*sup*_ = 2.43 V for *R*_2_ = 28 kΩ, and *V_L_*_,*inf*_ = 0.66 V and *V_H_*_,*sup*_ = 2.64 V for *R*_2_ = 40 kΩ. In the case of *V_t_* = *V_DD_*/2 = 1.65 V, the digital interface parameters are symmetrical about *V_DD_*/2, that is, *V_L_* − *V_L_*_,*inf*_ = *V_H_*_,*sup*_ − *V_H_* = 0.3 V. This results in a wide range of the duty cycle that is symmetrical about 50%. In the case of *V_t_* = 1.15 V, instead, it is *V_L_* − *V_L_*_,*inf*_ = 0.24 V and *V_H_*_,*sup*_ − *V_H_* = 1.24 V, thus the duty-cycle minimum value is comparable to the case *V_t_* = 1.65 V, while the duty-cycle maximum value is much lower, resulting in a reduced duty-cycle range. Similarly, in the case of *V_t_* = 2.15 V, instead, it is *V_L_* − *V_L_*_,*inf*_ = 1.24 V and *V_H_*_,*sup*_ − *V_H_* = 0.24 V; thus, the duty-cycle maximum value is comparable to the case *V_t_* = 1.65 V, while the duty-cycle minimum value is much higher, resulting in a reduced duty-cycle range.

**Figure 12:**
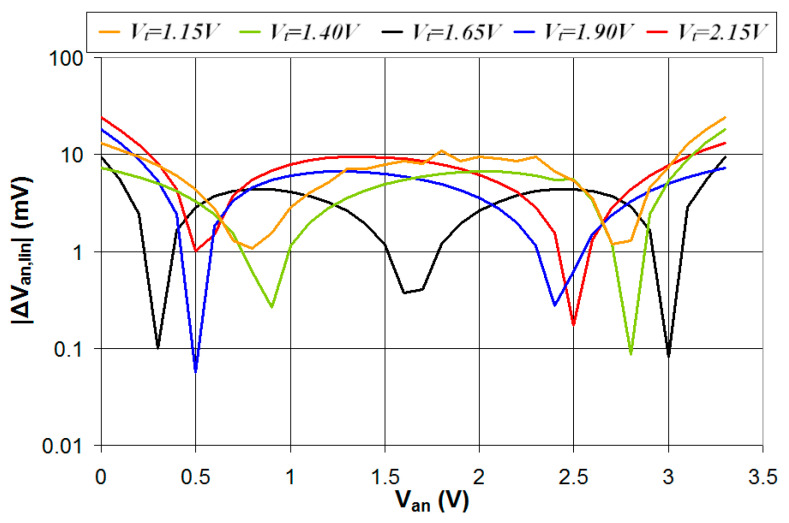
Absolute value of the error in the estimated *V_an_* plotted vs. the real value of *V_an_* for five different values of *V_t_* and Δ*V* = 0.5 V.

The analog voltage *V_an_* was estimated from the value of the duty cycle using the linear regression line fitting the curves presented in Figure 11, and the absolute value of the error in the estimated *V_an_* (|Δ*V_an_*_,*lin*_|) is plotted vs. *V_an_* for the five different values of *V_t_*. The results presented in Figure 12 show that the case *V_t_* = 1.65 V is characterized by a higher duty-cycle range and better linear response of the relation between the duty cycle and *V_an_*, thus resulting in a lower error in the estimated value of *V_an_*. This shows how *V_t_* = *V_DD_*/2 is the best condition that results in the maximization of the accuracy in the estimated analog voltage *V_an_*. 

### 3.4. Maximum Performance of the Proposed Method

In this section, the maximum performance of the proposed technique to estimate an analog voltage *V_an_* without the use of an ADC was evaluated. The performance was calculated in the ideal case of absence of noise, considering only the uncertainty in *t_H_* and *t_L_* due to the finite clock frequency. A case study is considered where the clock frequency of the device that implements the technique is set to 27 MHz (i.e., period of *T* = 37.037 ns) and *t_H_* and *t_L_* are measured using a 16 bit counter (i.e., maximum time interval 2.427 ms). Indicating that when *N_H_* and *N_L_* are the number of clock counts to measure *t_H_* and *t_L_*, respectively, the duty cycle of the signal *V*_2_ can be expressed as

(22) $$DC=100\frac{N_{H}T}{N_{H}T+N_{L}T}=100\frac{N_{H}}{N_{H}+N_{L}}$$

Considering the error propagation in a quotient, the error in the measured duty cycle is

(23) $$\Delta DC=100\Delta \left(\frac{N_{H}}{N_{H}+N_{L}}\right)=100\left[\frac{\Delta N_{H}}{N_{H}+N_{L}}+\frac{N_{H}}{{\left(N_{H}+N_{L}\right)}^{2}}\Delta \left(N_{H}+N_{L}\right)\right]$$

Since, in absence of noise, it is Δ*N_H_* = Δ*N_L_* = 1 and Δ(*N_H_ + N_L_*) = Δ*N_H_* + Δ*N_L_* = 2, the worst case (maximum ΔDC) is obtained when *N_H_* ≈ *N_H_* + *N_L_* (high values of duty cycle). It is

(24) $$\Delta DC_{\max}\approx \frac{300}{N_{H}+N_{L}}=300\frac{f_{clock}}{f_{PWM}}$$

where *f_clock_* is the frequency of the clock signal (27 MHz) and *f_PWM_* is the frequency of the signal *V*_2_. The maximum error of the measured duty cycle (Δ*DC_max_*) was calculated using Equation (24) and the error of *V_an_* due to the limited clock frequency and counter resolution (|Δ*V_an_*_,*clock*_|) estimated by considering the relation between the duty cycle and *V_an_* presented in Figure 5d, thus not considering the uncertainty due to the deviation of the characteristic from the regression line. The results are presented in Table 2 for different values of the resistance *R*_2_ and the capacitance *C*. In Table 3, the maximum sampling rate is presented for the same values of the resistance *R*_2_ and the capacitance *C*. As can be seen, the maximum error |Δ*V_an_*_,*clock*_| decreases with the increase of *C*, since higher values of the capacitance result in longer measured times, thus higher resolution due to the counter, and with the decrease of *R*_2_, since this results in a higher range of the duty cycle and, thus, higher resolution of the measured duty cycle. At the same time, however, lower values of the capacitance *C* allow a much higher sampling frequency. Thus, in a noisy environment, with low SNR values, taking measurements with a low value of *C* allows to take advantage of the higher sampling frequency by averaging the measurements on a large number of samples, thus reducing the uncertainty due to noise. 

By neglecting the error of *V_an_* due to the nonlinearity of the relation between *V_an_* and the duty cycle, for example, by mapping the values using a LUT on the device memory, an error for *V_an_* of approximately 0.67 mV (lower than a 12 bit ADC) can be achieved with a maximum sampling frequency of 863 Hz that is more than 10 times higher than the sample frequency reported in [41] (65 Hz). The maximum error for *V_an_* accounting also the error from linear modeling is obtained by adding |Δ*V_an_*_,*clock*_| with the error for *V_an_* due to modeling with the linear regression line (|Δ*V_an_*_,*lin*_|, i.e., data presented in Figure 8) and the results are presented in Table 4. In this case the value of *V_an_* is estimated from the measured duty cycle using a linear function, thus without a LUT to map the values of *V_an_* to the values of duty cycle. The minimum error for *V_an_* that can be achieved is 1.33 mV (close to the resolution of a 12 bit ADC) with a sampling frequency of 497 Hz.

## 4. Implementation in a FPGA Device

The proposed technique to estimate an analog voltage without the use of an ADC was implemented in a commercial FPGA, Gowin GW1NR-9 (Gowin Semiconductor, Guangzhou, China), integrated on a Tang nano 9k development board (Sipeed, Shenzhen, China) [47]. The Gowin GW1NR-9 is a low-cost FPGA that does not integrate an internal ADC for analog voltage measurements. 

**Figure 13:**
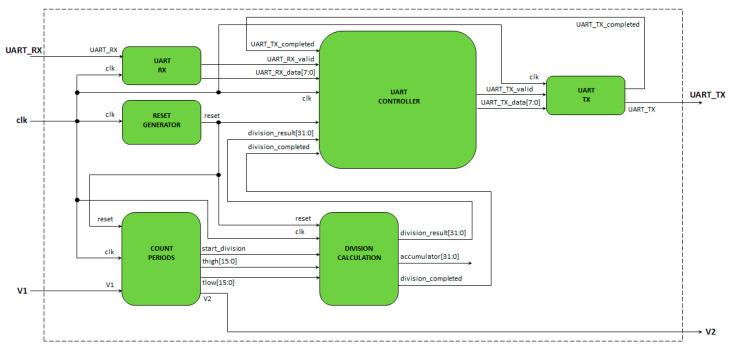
Schematic of the digital circuit for the estimation of an analog voltage without the use of an ADC.

The schematic of the digital circuit, designed in Verilog and implemented in the FPGA, is presented in Figure 13. The digital input *V*_1_ and digital output *V*_2_ are interfaced with the *RC* circuit as presented in Figure 1. The clock signal *clk* (27 MHz) is generated using the 27 MHz crystal oscillator present on the Tang nano 9k development board. The *UART_RX* and *UART_TX* signals are interfaced with the USB-UART connector of the development board to allow UART communication (baud rate 115200) with a PC using ad-hoc developed LabVIEW programs (National Instruments).

The working principle of the designed digital circuit is defined by the following steps:

(1) An 8 bit command is sent from the PC to the FPGA using UART to request the value of the duty cycle of signal *V*_2_.
(2) The FPGA module ‘count periods’ calculates the length of time signal *V*_2_ is high (*t_H_*) and low (*t_L_*) and stores these data in two 16 bit registers.
(3) The FPGA module ‘division calculation’ calculates the duty cycle of signal *V*_2_ from the 16 bit registers *thigh* and *tlow* and stores the result in the 32 bit register *division_result*.
(4) The measured duty cycle is sent from the FPGA to the PC using UART with four 8 bit data transfers.

**Figure 14:**
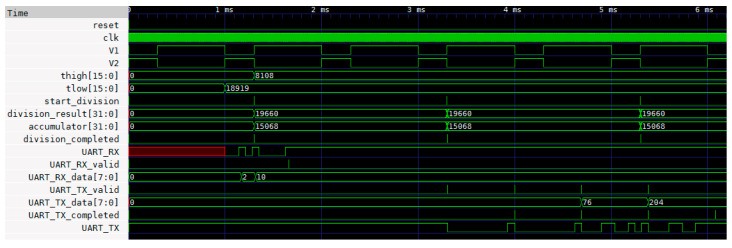
Simulation of the designed digital circuit for the estimation of an analog voltage without the use of an ADC.

Simulations of the Verilog code were carried out using the Icarus Verilog simulator [49] under Windows in the case of a *V*_2_ signal of frequency 1 kHz and a duty cycle of 30%. The waveforms of the signals are presented in Figure 14. The time intervals when *V*_2_ = *V_DD_* (*t_H_*) and *V*_2_ = 0 (*t_L_*) are measured by the ‘count periods’ module using a 16 bit counter and stored inside two 16 bit registers (*thigh* and *tlow*). After the two time intervals are measured, a pulse is generated on the *start_division_signal* to trigger the duty-cycle calculation. This is carried out by the ‘division calculation’ module that accepts as input the 16 bit data *thigh* and *tlow* and generates a 32 bit signal *division_result* that is proportional to the duty cycle. In particular, *division_result* is calculated as the binary division between two 32 bit registers: *A*, with the most significant 16 bits equal to *thigh* and the least significant 16 bits equal to 0, and *B*, with the most significant 16 bits equal to 0 and the least significant 16 bits equal to *thigh* + *tlow*. After the binary division, carried out using the Verilog implementation presented in [50], the division result is stored in the 32 bit register *division_result*, a pulse is generated on the signal *division_completed*, and the duty cycle can be calculated using the formula

(25) $$DC=100\frac{division\_result}{2^{16}}$$

The ‘UART controller’, ‘UART TX’, and ‘UART RX’ modules are used for the communication between the PC and the FPGA device. When the 8 bit decimal code 10 is received, the 32 bit register *division_result* is transferred to the PC with four 8 bit data transfer. The Verilog code of the proposed implementation on FPGA is available in the Supplementary Materials.

## 5. Experimental Results

**Figure 15:**
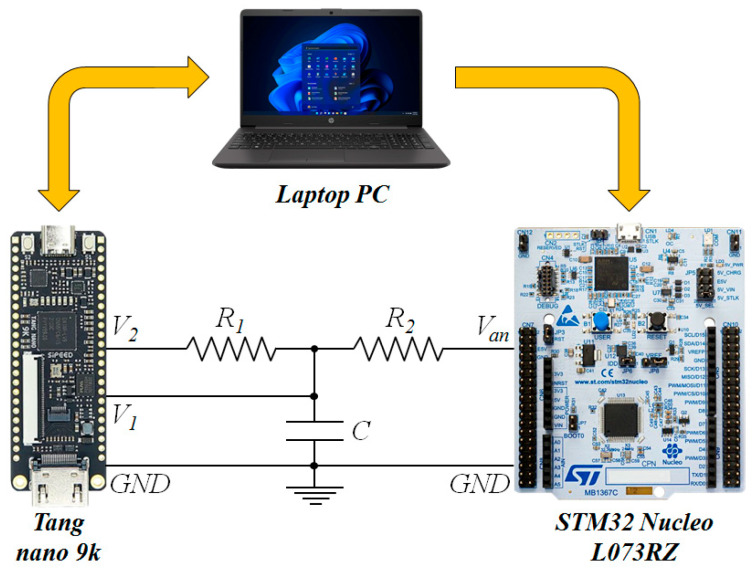
Measurement setup used to validate the proposed technique for analog voltage measurements without the use of an ADC.

The measurement technique, implemented on the Gowin GW1NR-9 FPGA device [51] integrated in the Tang Nano 9k development board [47], was tested using the measurement setup shown in Figure 15. After the place-and-route step, the FPGA resources used were 498/8640 of the programmable LUTs and ALUs (5%), 369/6693 of the registers (5%), 329/4320 of the configurable logic slice (7%), and 5 I/O ports. The analog voltage *V_an_* to be measured is generated using the 12 bit DAC integrated in the STM32 microcontroller present on the Nucleo-L073RZ development board. A Laptop PC is used to communicate with the microcontroller board and the FPGA board using the USB-UART interface. The PC sends a control message to the Nucleo-L073RZ board to set the value of the analog voltage *V_an_* at the output of the 12 bit DAC. Similarly, the PC sends a control message to the FPGA board (Tang nano 9k) to request the measured value of the duty cycle of the signal *V*_2_. The circuit composed of the resistances *R*_1_ and *R*_2_ and the capacitance *C* was implemented using discrete components mounted on a solderless breadboard.

First of all, the noise level of the circuit was evaluated by measuring the analog voltage *V_an_* using the 12 bit ADC integrated in the STM32 microcontroller. Seven different *V_an_* values were set with the 12-bit DAC: 0.419 V, 0.832 V, 1.244 V, 1.656 V, 2.068 V, 2.481 V, and 2.894 V.

For each *V_an_* value, 10,000 acquisitions were made using the 12 bit ADC and the statistical distribution of the acquired analog voltages evaluated. The average standard deviation (*σ*) of the analog voltage distribution was 4.442 mV. Thus, considering an uncertainty of ±2*σ*, the maximum error in the acquired analog voltage was 17.768 mV (confidence level 95%). This is significantly higher than the 12 bit ADC resolution (0.806 mV). The accuracy of the measured analog voltage *V_an_* can be increased by averaging different measurements. Indicating that when *N* is the number of measurements used to estimate *V_an_*, the standard deviation of the distribution of the analog voltage with *N* measurements is

(26) $$\sigma_{N}=\frac{\sigma }{\sqrt{N}}$$

To obtain the reading uncertainty typical of a 12 bit ADC (0.806 mV), it should be *σ_N_* = 0.2015 mV and, given *σ* = 4.442 mV, the number of measurements *N* should be 486. Thus, under the current noise level, the original 1 MHz ADC sampling rate must be reduced to approximately 2057 Hz to guarantee a measurement uncertainty of 0.806 mV for the analog voltage *V_an_*. 

**Figure 16:**
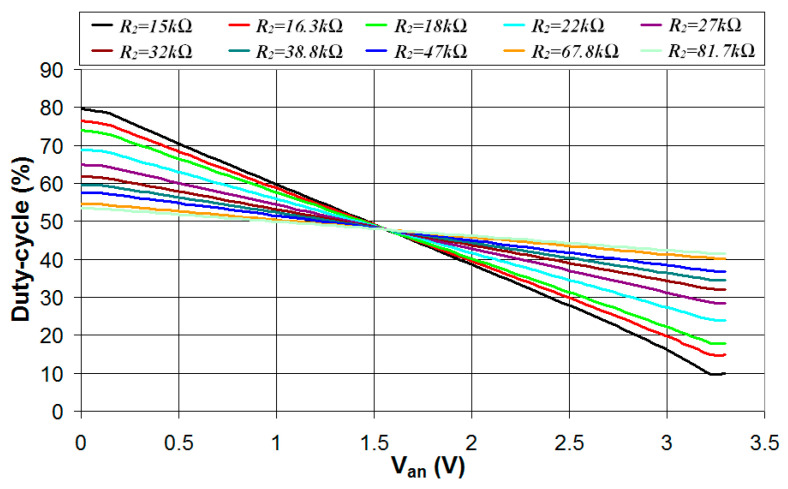
Measured duty cycle of the digital signal *V*_2_ plotted vs. the analog voltage *V_an_* for *R*_1_ = 10 kΩ, *C* = 1 nF, and different values of the resistance *R*_2_.

Using the measurement setup presented in Figure 15, the resistance *R*_1_ was set to 10 kΩ and 10 different values of the resistance *R*_2_ were tested (15 kΩ, 16.3 kΩ, 18 kΩ, 22 kΩ, 27 kΩ, 32 kΩ, 38.8 kΩ, 47 kΩ, 67.8 kΩ, and 81.7 kΩ). For each value of the resistance *R*_2_, 10 different values of the capacitance *C* were tested (1 nF, 2 nF, 4.9 nF, 9 nF, 14 nF, 20 nF, 32 nF, 47 nF, 62 nF, 106 nF). Each measurement was carried out on 83 different *V_an_* values distributed on the 0–3.3 V range (steps of 39.759 mV), and, for each *V_an_* value, 1000 acquisitions were carried out to evaluate the average value and the standard deviation of the estimated *V_an_*. The results showed no significant difference in the standard deviation of the estimated *V_an_* for different values of the capacitance *C*. This can be explained by the high levels of noise introduced by the circuit implementation on a solderless breadboard with fly wires. Thus, in the following, the experiments are presented for the capacitance *C* = 1 nF since this results in the highest sampling rate and allows averaging of the measured signal on a larger number of samples to reduce the error. In Figure 16, the measured duty cycle plotted vs. the analog voltage *V_an_* is presented for different values of *R*_2_ and *C* = 1 nF.

As can be seen, the duty cycle presents a very good linear relationship with the analog voltage *V_an_* in the range 0.12–3.22 V. The saturation of the characteristic outside this range was observed also in the measurements of *V_an_* using the microcontroller integrated 12 bit ADC and can be explained by a saturation of the output of the 12 bit DAC used to generate *V_an_*. Thus, in the following, the performance of the technique deployed on the FPGA device was evaluated in the *V_an_* range 0.12–3.22 V. For each value of the resistance *R*_2_, the linear regression equation that best fits the characteristic duty cycle *V_an_* was calculated offline using a PC. The calculated slope and offset of the linear regression line were stored in the FPGA memory and used to estimate the value of the analog voltage *V_an_*. The maximum deviation to the real value of *V_an_* (|Δ*V_an_*|) was also evaluated. The measured standard deviation (*σ*) of the estimated *V_an_* was considered for the determination of the number of measurements (*N*) needed to obtain the reported accuracy in the *V_an_* estimation, and this value was used to calculate the maximum sampling rate (*SR_max_*). The results are reported in Table 5. As can be seen, the uncertainty in the analog voltage estimation (|Δ*V_an_*|) presents a minimum for *R*_2_ = 32 kΩ. This is the result of two different effects: (a) lower values of *R*_2_ are characterized by a higher range of the duty cycle and, thus, higher sensitivity in the *V_an_* estimation (this is confirmed by the higher standard deviation *σ* in the case of higher values of *R*_2_) and (b) higher values of *R*_2_ result in stronger linearity of the duty-cycle *V_an_* characteristic, thus lower error in the estimation of *V_an_* using the linear regression line. In the end, the highest accuracy in the *V_an_* estimation (1.09 mV) is obtained for *R*_2_ = 32 kΩ with a maximum sampling rate of 9.75 Hz. Under these conditions, the analog voltage is measured with an effective number of bits ENOB = 10.82, a signal-to-noise ratio SNR = 66.9 dB, a differential nonlinearity DNL = 0.12 LSB, and an integral nonlinearity INL = 0.72 LSB. The maximum sampling rate can be increased to 31.35 Hz and 128.31 Hz with a limited reduction in the estimated *V_an_* accuracy (maximum errors of 1.61 mV and 2.68 mV, respectively). This was achieved using a solderless breadboard implementation with fly wires. The implementation on a custom designed electronic board integrating the FPGA and the measurement circuit is expected to significantly reduce the noise level and thus improve the measurement accuracy. 

## 6. Comparison with the State of the Art

The performance of the proposed technique for analog voltage measurement without an ADC was compared to similar techniques from the literature, as well as a 12 bit ADC integrated in a low-cost microcontroller, and the results are presented in Table 6.

The Nucleo-L073RZ is a development platform with onboard the microcontroller STM32L073RZT6 by ST-Microelectronics that integrates a 12 bit ADC [43]. This is a very popular platform for the development of portable battery-operated sensor systems due to the low power consumption. The internal 12 bit ADC works in the range 0–3.3 V with a maximum error (in the ideal case of absence of noise) of 0.806 mV and a sampling rate of 1 MHz.

The technique proposed by Peter et al. in 1998, implemented on the PIC16C6XX series of microcontrollers, presents a 10 bit delta-sigma ADC using the analog comparator integrated in the microcontroller and a digital filter [37]. The measured analog voltages are in the range 0–5 V, the reported effective resolution is 8 bit (maximum error 19.53 mV), and a single measurement takes 20.48 ms (maximum sample rate 48.83 Hz).

The work by Soldera et al. also implements a 10 bit delta-sigma ADC on the HC9S08Rx family of microcontrollers working at 8 MHz and exploits the analog comparator integrated in the microcontroller for the analog voltage measurements in the range 0–3.3 V [38]. The reported maximum error is 3.22 mV with a sampling rate of 125 Hz.

The technique presented by Weber and Windish in 2007 implements an analog voltage measurement without an ADC using the microcontroller MSP430F2274 (Texas Instruments) [39]. The proposed implementation is suited for the measurement of control signals in an industrial environment (0–10 V, 0–20 mA) and uses an external AD7400 delta-sigma modulator (Analog Devices). No data are reported about the measurement accuracy and sampling rate.

A delta-sigma modulator for analog voltage measurements was also proposed in 2011 with an implementation on an Altera Cyclone IV FPGA device [40]. The reported sampling rate is very high (500 kHz), with a maximum error for the measured voltage of 3.22 mV. However, the proposed technique exploits the Low Voltage Differential Signaling (LVDS) receiver integrated in the FPGA and, thus, can be implemented only on devices with this feature.

Another delta-sigma modulator on FPGA is presented by Lattice Semiconductor and implemented using a LVDS receiver or an external analog comparator for the FPGA devices that lack this feature [52]. The range of the analog voltage is 0–3.3 V, and the reported maximum error is 12.88 mV with a sampling rate of 7.63 kHz.

Differently from the previous implementations presented in [37,38,39,40,52] that use an analog comparator, either integrated in the device or external, the works proposed by Bengtsson in 2012 exploit the Schmitt trigger circuit of the digital input port of a PIC18F458 microcontroller for the voltage measurements [41]. The measurement range is 0–5 V, with a reported maximum error of 1.22 mV and a sampling frequency of 65 Hz. To achieve this measurement accuracy, a LUT, stored in the microcontroller memory, is used to map the time periods measured with the internal counter to the analog voltage values.

The presented work implements an analog voltage measurement without an ADC in a low-cost FPGA device (Gowin GW1NR-9). The novelty of the proposed solution lies in the exploitation of the Schmitt trigger circuit of the digital input pin of a microcontroller or FPGA combined with the estimation of the analog voltage from the duty cycle of a square-wave signal. The exploitation of the Schmitt trigger circuit of the digital input pin allows avoidance of the use of an analog comparator while the property of the duty cycle of the monitored waveform signal to feature a good linear correlation with the analog voltage is exploited to avoid the use of a LUT, with benefits in terms of lower memory occupation. The works in [37,38,39,40,52] propose delta-sigma ADCs implemented in different microcontrollers and FPGAs that make use of an analog comparator and a reference voltage. The works in [40,52] remove the need for an analog comparator by the use of a LVDS receiver, but this high-speed differential signal technology is generally not integrated in microcontrollers and low-cost FPGAs. The work of Bengtsson [41], instead, exploits the Schmitt trigger property of the digital input pin of a microcontroller like the proposed solution, but estimates the analog voltage with a nonlinear characteristic and using a LUT to map the measured counter time to the analog voltage, with increased cost in terms of memory occupation. The proposed solution features a maximum error in absence of noise of 1.33 mV with a sampling frequency of 497 Hz. The results from experimental measurements in a noisy environment show that an accuracy close to a 12 bit ADC can be achieved with a sampling frequency of 9.75 Hz or, alternatively, a maximum error of 1.61 mV or 2.68 mV for a sampling frequency of 31.35 Hz and 128.31 Hz, respectively.

## 7. Conclusions

A novel technique was presented to implement a direct interface between a sensor with analog output voltage and the digital input ports of a microcontroller or Field-Programmable Gate Array (FPGA). The proposed method requires, in its simplest implementation, only a few discrete passive components (two resistors and a capacitor) and estimates the analog voltage from the duty cycle of a square-wave digital signal using a quasi-linear characteristic. Simulations using LTSpice have shown that, using a 16 bit counter, a resolution of 1.33 mV can be achieved with a maximum sampling rate of 497 Hz and without the use of look-up tables to map the analog voltages to the duty-cycle values, thus with significant reduction in memory occupation. The proposed technique was implemented in a low-cost commercial FPGA using a solderless breadboard with fly wires, and the test results showed a maximum error of 1.09 mV with a sampling rate of 9.75 Hz. The sampling rate can be increased with a limited reduction in the analog voltage estimation accuracy (31.35 Hz for a maximum error of 1.61 mV and 128.31 Hz for a maximum error of 2.68 mV). A potential issue that can impact the accuracy of the proposed technique is the variation of the interface parameters with environmental conditions (temperature and humidity) and power supply, as well as with the aging of the device. Future investigations in this research line will be aimed at evaluating these effects and proposing countermeasures to mitigate the impact on the measurement accuracy.

## Figures and Tables

**Table 1:** Characteristics of the digital interface parameters for different microcontrollers and FPGAs.

Device	Device Type	Development Board	Ref.	*V_H_* (V)	*V_L_* (V)	*V_t_* (V)	Δ*V* (mV)	*CV*_Δ*V*_
STM32L073RZT6	microcontroller	Nucleo-L073RZ	[43]	1.644	1.196	1.420	448	1.827
Atmel SAM3X8E	microcontroller	Arduino Due	[44]	1.481	1.272	1.376	209	4.877
XC7A35T-1CPG236C	FPGA	Basys 3	[45]	1.281	1.228	1.254	52	6.527
XC7Z010-1CLG400C	FPGA	Zybo	[46]	1.337	1.239	1.288	97	1.425
Gowin GW1NR-9	FPGA	Tang Nano 9k	[47]	1.753	1.387	1.570	367	1.558

**Table 2:** Maximum error of *V_an_* (in mV) due to the limited clock frequency and counter resolution for different values of the resistance *R*_2_ and the capacitance *C*. Color-map: green—10th percentile, yellow—50th percentile, and red—90th percentile.

	*R*_2_ (kΩ)									
	120	62	35	27	22	18	17	16	15	14
**C = 320 nF**	2.21	1.12	0.59	0.42	0.31	0.22	0.19	0.17	0.14	0.10
**C = 150 nF**	4.71	2.36	1.25	0.90	0.67	0.47	0.41	0.35	0.29	0.22
**C = 60 nF**	11.77	5.91	3.13	2.25	1.67	1.17	1.03	0.89	0.73	0.55
**C = 47 nF**	15.03	7.54	3.99	2.88	2.14	1.49	1.32	1.13	0.93	0.70
**C = 32 nF**	22.09	11.01	5.86	4.23	3.14	2.19	1.93	1.66	1.37	1.03
**C = 20 nF**	35.31	17.72	9.38	6.76	5.02	3.51	3.09	2.66	2.19	1.64
**C = 14 nF**	50.44	25.32	13.40	9.66	7.18	5.01	4.42	3.80	3.12	2.35
**C = 9 nF**	78.47	39.39	20.85	15.03	11.17	7.79	6.88	5.91	4.86	3.66
**C = 5 nF**	141.25	70.89	37.53	27.06	20.10	14.03	12.38	10.63	8.74	6.58
**C = 2 nF**	353.12	177.24	93.83	67.65	50.26	35.07	30.94	26.58	21.86	16.46
**C = 1 nF**	706.30	354.53	187.67	135.30	100.52	70.15	61.89	53.12	43.72	32.92

**Table 3:** Maximum sampling frequency (in Hz) for different values of the resistance *R*_2_ and the capacitance *C*. Color-map: green—10th percentile, yellow—50th percentile, and red—90th percentile.

	*R*_2_ (kΩ)									
	120	62	35	27	22	18	17	16	15	14
**C = 320 nF**	510	497	469	441	404	349	327	300	266	218
**C = 150 nF**	1089	1062	1002	941	863	744	698	641	568	465
**C = 60 nF**	2724	2655	2505	2353	2159	1861	1747	1604	1420	1163
**C = 47 nF**	3478	3389	3197	3004	2757	2376	2230	2048	1812	1485
**C = 32 nF**	5113	4978	4696	4412	4049	3491	3275	3008	2662	2182
**C = 20 nF**	8174	7965	7515	7060	6479	5585	5241	4813	4260	3491
**C = 14 nF**	11,678	11,379	10,736	10,086	9256	7979	7487	6875	6086	4988
**C = 9 nF**	18,165	17,701	16,700	15,690	14,398	12,412	11,647	10,695	9467	7759
**C = 5 nF**	32,698	31,862	30,060	28,242	25,916	22,342	20,964	19,252	17,042	13,967
**C = 2 nF**	81,746	79,655	75,154	70,606	64,792	55,856	52,413	48,132	42,605	34,919
**C = 1 nF**	163,505	159,337	150,308	141,203	129,584	111,719	104,832	96,190	85,207	69,842

**Table 4:** Maximum error of *V_an_* (in mV) due to the limited clock frequency, counter resolution and the linearity approximation for different values of the resistance *R*_2_ and the capacitance *C*. Color-map: green—10th percentile, yellow—50th percentile, and red—90th percentile.

	*R*_2_ (kΩ)									
	120	62	35	27	22	18	17	16	15	14
**C = 320 nF**	2.25	1.33	1.55	2.44	4.21	8.53	10.85	14.33	19.99	29.45
**C = 150 nF**	4.75	2.58	2.21	2.92	4.57	8.78	11.07	14.51	20.14	29.57
**C = 60 nF**	11.81	6.13	4.09	4.27	5.57	9.48	11.69	15.05	20.58	29.90
**C = 47 nF**	15.07	7.76	4.95	4.90	6.04	9.80	11.98	15.29	20.78	30.05
**C = 32 nF**	22.13	11.30	6.82	6.25	7.04	10.50	12.59	15.82	21.22	30.38
**C = 20 nF**	35.35	17.94	10.34	8.78	8.92	11.81	13.75	16.82	22.04	30.99
**C = 14 nF**	50.49	25.54	14.36	11.68	11.08	13.32	15.08	17.96	22.97	31.70
**C = 9 nF**	78.51	39.61	21.81	17.05	15.07	16.10	17.54	20.07	24.71	33.00
**C = 5 nF**	141.29	71.11	38.49	29.08	24.00	22.34	23.04	24.79	28.59	35.93
**C = 2 nF**	353.16	177.46	94.79	69.67	54.16	43.38	41.60	40.74	41.71	45.81
**C = 1 nF**	706.34	354.75	188.63	137.32	104.42	78.46	72.55	67.28	63.57	62.27

**Table 5:** Performance of the proposed technique to estimate an analog voltage implemented in a FPGA device.

*R*_2_ (kΩ)	|Δ*V_an_*| (mV)	*σ* (mV)	*N*	*SR_max_* (Hz)
15	14.57	16.18	20	7679.37
16.3	7.44	18.06	94	1680.08
18	5.69	19.82	194	847.50
22	2.68	24.64	1352	128.31
27	1.61	30.37	5708	31.35
32	1.09	37.22	18,665	9.75
38.8	1.18	43.97	22,074	8.36
47	1.46	53.42	21,510	8.66
67.8	2.19	77.13	19,901	9.45
81.7	2.63	93.02	20,064	9.40

**Table 6:** Performance comparison of the proposed technique for analog voltage measurements without an ADC with similar techniques from literature and a 12 bit ADC integrated in a low-cost microcontroller.

ADC Used	Device Type	Comparator Used	Measurement Range	Maximum Error	Maximum Sample Rate	LUT Used	Ref.
Yes	μcontroller	No	0–3.3 V	0.81 mV	1 MHz	No	[43]
No	μcontroller	Yes	0–5 V	19.53 mV	48.83 Hz	No	[37]
No	μcontroller	Yes	0–3.3 V	3.22 mV	125 Hz	No	[38]
No	μcontroller	Yes	0–10 V	−	−	No	[39]
No	FPGA	Yes	0–3.3 V	3.22 mV	500 kHz	No	[40]
No	FPGA	Yes	0–3.3 V	12.88 mV	7.63 kHz	No	[52]
No	μcontroller	No	0–5 V	1.22 mV	65 Hz	Yes	[41]
No	FPGA	No	0–3.3 V	1.33 mV	497 Hz	No	This work

## Data Availability

Data are contained within the article and Supplementary Materials.

## Supplementary Materials

The following supporting information can be downloaded at: https://www.mdpi.com/article/10.3390/s24030873/s1.
